# Days Needed to Characterize the Healthfulness of a Typical Dinner Meal in Direct Observational Research: Mixed Methods Study

**DOI:** 10.2196/22541

**Published:** 2021-03-24

**Authors:** Allan Tate, Amanda Trofholz, Michael Miner, Jerica Berge

**Affiliations:** 1 Department of Epidemiology and Biostatistics College of Public Health University of Georgia Athens, GA United States; 2 Department of Family Medicine and Community Health University of Minnesota Medical School University of Minnesota Minneapolis, MN United States

**Keywords:** meal healthfulness, direct observation, family meals, well-being, diet, food

## Abstract

**Background:**

Prior research around the home meal environment has demonstrated that family meals are associated with positive health outcomes for children and adolescents. Researchers have begun using direct observational methods to understand key aspects of family meals such as meal healthfulness and family meal frequency to explain the protective nature of family meals. Direct observational research, however, can be resource intensive and also burdensome for participants. Information about the number of days needed to sufficiently characterize typical meal healthfulness using direct observational research methods is needed.

**Objective:**

The current study aimed to produce guidance about the number of meals necessary to approximate typical meal healthfulness at the family dinner meal occasion in a direct observational, mixed methods study of the home food environment.

**Methods:**

Families were recruited between 2012-2013 from primary care clinics in the Minneapolis–St Paul metropolitan area (N=120). A total of 800 meals were collected as part of the Family Meals LIVE! mixed methods study. The Healthfulness of Meal Index was used to evaluate meal dietary healthfulness of foods served at 8 family meal occasions. Participating families were provided an iPad (Apple Inc) and asked to video-record 8 consecutive days of family dinner meals with a minimum of two weekend meals. After the meal, families completed a meal screener, which is a self-reported, open-ended measure of the foods served at the meal.

**Results:**

Weekend and weekday meals differed in their measurement of meal healthfulness, indicating that at least one weekday and one weekend day are necessary to approximate meal healthfulness. Single-day measurement mischaracterized the strength of the relationship between the quality of what was served and intake by almost 50%, and 3 to 4 observation days were sufficient to characterize typical weekly meal healthfulness (*r*=0.94; *P*<.001).

**Conclusions:**

Relatively few direct observational days of family meals data appear to be needed to approximate the healthfulness of meals across 1 week. Specifically, 1 weekday and 1 weekend observation are needed, including a total of 3 to 4 days of direct observational meal data. These findings may inform future direct observational study designs to reduce both research costs and participant burden in assessing features of the meal environment.

## Introduction

Having frequent family dinner meals has consistently been associated with a number of beneficial health outcomes for children, including reduced risk of being overweight [1-3] and healthy diet quality [4-10]. Additionally, quality of the emotional atmosphere [11,12] during family meals and quality of the food served during these meals [13] have been previously characterized as pathways that affect child weight and health outcomes. Direct observational research methods (ie, video recording) are becoming more common in family meals research because they overcome the reporting bias found in commonly used survey-based measures, allowing for a more in-depth and robust picture of the characteristics (eg, interpersonal interactions, meal healthfulness) of family meals that may contribute to child and adolescent health [11,14]. However, the impact of both the timing of the direct observational measurement and duration of the observational measurement period on estimates of meal healthfulness have not been examined.

In the current methodological study, the Healthfulness of Meal Index (HOM), implemented in the Family Meals LIVE! direct observational study [15], was used to answer the research question: how many days of direct observation of the foods served at family dinner meals are needed to characterize “typical” healthfulness of the meal to preserve resources and reduce participant burden? Family dinner meals were defined as an evening meal eaten in the home environment with the majority of family members present. The study further examined if weekends and weekdays influence meal healthfulness and at what number of days the addition of an observation day becomes unnecessary to characterize relationships with child dietary intake. We hypothesized that weekday and weekend day meal healthfulness estimates would differ due to changes in the home meal environment when children are not at school or when parents are not generally at work. We also hypothesized that estimates incorporating fewer days of observations would be weakly correlated with estimates derived from a full week of dinner meals. Results of the current study address a salient public health nutrition research need of providing pragmatic design guidance that could result in improved measurement.

## Methods

### Sample Population

Data collected from Family Meals LIVE! [15], a direct observational, mixed methods study, were used to measure the healthfulness of foods served at 8 meal occasions. The University of Minnesota’s Institutional Review Board Human Subjects Committee approved the study protocol. Families (N=120) were recruited between 2012 and 2013 from 4 primary care clinics in the Minneapolis–St Paul metropolitan area that serve a racially/ethnically diverse, urban population of primarily low-income families. Participating families were provided an iPad (Apple Inc) and asked to video-record 8 consecutive days of family dinner meals with a minimum of two weekend meals. Only dinner meals in the home were recorded because of privacy issues. At the start of each meal, families spoke into the camera to indicate what foods were being served. After the meal, families completed a meal screener, which is a self-reported, open-ended measure of the foods served at the meal. Comprehensive study procedures have been described elsewhere [11,13].

In total, 800 meals were available for analysis [13]. Families were asked to record meal occasions over consecutive days, and recordings were taken every 1.8 days on average (SD 0.89), indicating good participant compliance with data collection procedures and minimal lack of family meals or meals outside of the home. A 1-day washout period was employed to allow families to acclimate to the study procedures and recording equipment.

### Direct Observational Research

Previous studies have shown that direct observational research conducted in the home using unstructured observations (eg, play, routines) has more predictive validity and reliability compared to laboratory settings using structured observations (eg, tasks given to participants) and allows participants to acclimate and exhibit less reactivity [16-18]. The lengthened, 8-day observation window has been shown to offer advantages over cross-sectional designs, which include the measurement of weekday and weekend meals, the capture of variability in the healthfulness of weekly meals, and more reliable and objective measurement of family meal occasions [16-18].

### Healthfulness of Meal Index

The HOM, created for the Family Meals Live! study and adapted from the Healthy Eating Index 2010 [19], was used to assess family meal healthfulness [13,15,20]. The HOM assesses 7 categories of foods served at meals: fruit, vegetables, dark green vegetables, dairy, protein, high sodium foods (reverse scored), and added sugars (reverse scored). A present-or-absent format is used to score the HOM, the components are summed, and a total of 9 points are available (the fruit and vegetable categories can each receive a total of 2 points). A higher total score is reflective of a more healthful family meal with regards to foods served. To calculate the HOM score, 3 research members (including 2 registered dietitians) watched each video-recorded meal to code the foods present [13]. The self-report meal screener was also used to corroborate the foods seen in the videos. Because the HOM evaluates meal dietary healthfulness, all foods present were coded even if they were not consumed by all family members.

### Meal Healthfulness Permutation Measures

Permutations were constructed to evaluate study conditions (timing of measurement and duration of measurement period) that researchers implemented at the design stage of direct observational studies. First, a permutation was calculated to examine how adding observation days affects the HOM relative to a measure that incorporates all observation days. In all, 13 HOM permutations were calculated: a full-week index of average meal healthfulness (this was the primary reference permutation), 6 indices adding 1 additional day on the front end of the observation window (permutation 1: day 1 only; permutation 2: average of days 1 and 2; permutation 3: average of days 1 through 3; etc.), and 6 permutations adding 1 additional day beginning with the last observation day (measure 1: day 7 only; measure 2: average of days 7 and 6; measure 3: average of days 7, 6, and 5 etc). The primary reference permutation was computed assuming that capturing more dinner meals would reduce the random variation in the composition of foods that are served across days to obtain a measure of typical meal healthfulness. Relative to this comprehensive direct assessment of meal healthfulness, a measure containing fewer observation days that is highly correlated with the full measure may sufficiently characterize typical family meal healthfulness without excess resource investment.

### Statistical Analysis

Survey estimation procedures were performed for each permutation of the HOM to determine whether the means differed by day of week, with sampling weights being applied to obtain population average meal healthfulness measures generalizable to the 4 clinics from which families were recruited. Effect consistency in the relationship between the HOM and dietary intake and family meal frequency were examined in sensitivity analyses to evaluate the presence of measurement error in permuted variables with a fewer number of observation days. A third correlational analysis was performed to evaluate the strength of the linear relationship between each HOM permutation. Comparisons between each reduced measure and the full reference measure were examined to determine how many days of additional meal recordings were needed to approximate the full reference measure. The intraclass correlation coefficient (ICC; 0.663) was calculated to evaluate consistency across the permutations within families. Pearson correlation coefficients above the ICC were used to visually evaluate at what points the permutations with fewer measurement days approximated the measure incorporating all days. All analysis and data management were performed in Stata 13.1 SE (StataCorp).

## Results

The coefficient of variation for the single-day estimate of meal healthfulness was 39.3% (mean 3.3, SD 1.3) and declined to 27.2% as days were added to compute the full reference measure containing all observation days (mean 3.2, SD 0.9). Adding observation days increased the precision of the sample measure, and dispersion around the mean stabilized when 3 observation days were included. The full permutation was overall similar for weekend days (mean 3.1, SD1.4) compared with only weekday observations (mean 3.2, SD 0.9). The permutation variables (day 1 and day 7) which corresponded to measures that would be derived from a 1-day, cross-sectional study design indicated that weekend meal healthfulness was higher in one weekday contrast and less healthy in the other weekday contrast. An evaluation of the noncompliance pattern indicated that meal healthfulness became increasingly difficult to ascertain for more than five meals for the total sample. Specifically, 96.7% of the sample (116/120) provided enough meal recordings to calculate the 5-7–day meal healthfulness permutation, and 78.3% of the sample (94/120) provided a final meal (seventh meal) recording needed to calculate the final meal healthfulness permutation.

The relationships between quality of foods served, dietary intake, and frequency of family meals were examined. The dietary intake association was strongly attenuated when fewer observation days were used to estimate meal healthfulness (Table 1). Compared to the association observed when 4 days were used to compute meal healthfulness, the single-day measure of association was –48% weaker. By 4 days, the observed relationship between meal healthfulness and dietary intake was consistent with associations that included additional observation days. There was no evidence that the association between meal healthfulness and family meal frequency was strengthened or weakened according to how many meal healthfulness observation days were used. There was some evidence that inference would differ when adding observation days (ie, the statistical significance was not met at a *P* value of <.05).

Permutations of HOM were calculated by averaging the HOM scores calculated using 1 to 7 direct observation days. The bivariate associations between each permuted score and the Healthy Eating Index 2010 were examined. Increasing the number of direct observation days used to characterize the healthfulness of foods served (HOM) was positively correlated with healthy dietary intake of the participant child for all permutations (7-day permutation *P*=.001; Table 1). The magnitude of the associations grew as more observation days were included, and they remained similar after 3 or 4 observation days were added, suggesting that about 4 observation days may be sufficient to characterize how the healthfulness of food served at meals is related to child dietary intake.

Effect sizes expressed as correlation coefficient *r* were examined to evaluate the strength of the linear relationship between the permutations using fewer than 7 observation days and the permutation incorporating all observed meals over the observation period (Table 2). Results indicated that the linear relationship between measures (starting with a single day and adding additional days) grew stronger as more observation days were added. A second analysis (removing the first observation day until only the last observation day was used) indicated a consistent pattern. Meals occurring farther apart (ie, the day 1 permutation and the day 7 permutation, each of which use a single observation day), were weakly correlated (*r*=0.36), indicating meal healthfulness may vary across time. Permutations calculated from days closer together were strongly related (day 1 permutation and the permutation including both day 1 and day 2: *r*=0.80; permutation including day 6 and 7 and the day 7 permutation: *r*=0.82). The within-family ICC of all 13 permutations was moderate to strong (ICC 0.663), indicating moderate variation in family meal healthfulness. Four observation days sufficiently characterized the typical weekly meal healthfulness observed in the full measure (*r*=0.94).

**Table 1:** Association between the number of direct observation days in the healthfulness of meal index permutation and the Healthy Eating Index 2010 and weekly family meal frequency: (N=120) households (caregivers and children) recruited from Minneapolis–St Paul primary care clinics between 2012 and 2013.

Number of HOM^a^ permutation observation days	Healthy Eating Index 2010		Weekly family meal frequency	
	Mean response (95% CI)	*P* value	Mean response (95% CI)	*P* value
1 day	1.4 (0.12 to 2.61)	*.03* ^b^	0.3 (0.03 to 0.61)	*.03*
2 days	1.9 (0.39 to 3.39)	*.01*	0.4 (0.07 to 0.75)	*.02*
3 days	2.3 (0.74 to 3.77)	*.004*	0.3 (–0.04 to 0.66)	.08
4 days	2.6 (1.15 to 4.13)	*.001*	0.3 (–0.06 to 0.68)	.10
5 days	2.4 (0.86 to 3.84)	*.002*	0.2 (–0.11 to 0.58)	.18
6 days	2.5 (0.97 to 4.01)	*.002*	0.3 (–0.09 to 0.62)	.14
7 days	2.6 (1.05 to 4.07)	*.001*	0.3 (–0.04 to 0.68)	.08

^a^HOM: Healthfulness of Meal Index.

^b^Numbers in italics indicate significance at a *P* value <.05.

**Table 2:** Family meal healthfulness permutation measures with pairwise Pearson correlations. Correlation coefficients *r* are all significant at *P*<.001.

Permutation variable	Day 1, *r*	Days 1-2, *r*	Days 1-3, *r*	Days 1-4, *r*	Days 1-5, *r*	Days 1-6, *r*	All Days, *r*	Days 2-7, *r*	Days 3-7, *r*	Days 4-7, *r*	Days 5-7, *r*	Days 6-7, *r*	Day 7, *r*
Day 1	—^a^												
Days 1-2	0.80	—											
Days 1-3	0.70	0.90	—										
Days 1-4	0.64	0.83	0.93	—									
Days 1-5	0.60	0.78	0.90	0.97	—								
Days 1-6	0.57	0.76	0.89	0.95	0.98	—							
All days	0.57	0.76	0.88	0.94	0.96	0.98	—						
Days 2-7	0.37	0.63	0.80	0.88	0.92	0.95	0.97	—					
Days 3-7	0.34	0.48	0.70	0.81	0.87	0.90	0.93	0.96	—				
Days 4-7	0.34	0.46	0.56	0.72	0.80	0.84	0.88	0.90	0.94	—			
Days 5-7	0.33	0.48	0.59	0.62	0.73	0.80	0.86	0.88	0.89	0.92	—		
Days 6-7	0.31	0.46	0.52	0.52	0.52	0.65	0.73	0.74	0.74	0.76	0.86	—	
Day 7	0.36	0.43	0.43	0.44	0.45	0.45	0.61	0.61	0.61	0.67	0.72	0.82	—

^a^Not applicable.

## Discussion

### Principal Findings

Study results were consistent with our hypothesis that a fewer number of direct observation days would be sufficient to characterize typical weekly meal healthfulness. We also found evidence that including both weekday and weekend day family dinner meals differed in healthfulness across a week-long observation period. Single-day and 2-day observations of meal healthfulness may be inappropriate for generalizing about the healthfulness of foods served at dinner meal occasions over the course of a week. In addition, correlational analyses indicated that when using just 2 days of data, the fewer-day permutations were strongly correlated (*r*>0.70) with the full 7-day measure. This is in part because meal healthfulness was moderately to highly correlated within the family. Thus, it is not surprising that adding a fourth, fifth, and sixth day of observational data provided little additional information about the healthfulness of foods served. Using 3- or 4-day observations of family meal healthfulness appeared to maximize measurement reliability and to minimize the cost of data collection and respondent burden.

### Study Limitations and Strengths

The study had several strengths, including the use of direct observational methods, consecutive observation of family meals, and a substantial number of meals (N=800) observed. Practical advantages are also noted, such as assessing measurement variability, providing new information about how to allocate staff time, and minimizing respondent burden. Replication studies are needed to provide support for the finding that relatively few observation days (ie, 1 weekend day and 1 weekday) are required, with the ideal number of days possibly being as few as 4; to test findings in a population with heterogeneous characteristics; and to assess meal healthfulness in multiple ways to avoid social desirability bias, recall error, and participant reactivity.

### Conclusions

Findings from the current study suggest that relatively few direct observational days of family meals data are needed to approximate the healthfulness of meals across 1 week. Specifically, 1 weekday and 1 weekend observation at a minimum, along with 3-4 days of direct observational data, are needed. Findings from the current study may inform future direct observational study designs to reduce both research costs and participant burden.

## Abbreviations

HOM: Healthfulness of Meal Index
ICC: intraclass correlation coefficient
